# The Effect of Dried *Glycyrrhiza Glabra L.* Extract on Obesity Management with Regard to PPAR-γ2 (Pro12Ala) Gene Polymorphism in Obese Subjects Following an Energy Restricted Diet

**DOI:** 10.15171/apb.2017.027

**Published:** 2017-06-30

**Authors:** Nazli Namazi, Mohammad Alizadeh, Elham Mirtaheri, Safar Farajnia

**Affiliations:** ^1^Diabetes Research Center, Endocrinology and Metabolism Clinical Sciences Institute, Tehran University of Medical Sciences, Tehran, Iran.; ^2^Nutrition Research Center, Faculty of Nutrition, Tabriz University of Medical Sciences, Tabriz, Iran.; ^3^Drug Applied Research Center, Tabriz University of Medical Sciences, Tabriz, Iran.

**Keywords:** Licorice, Hypocaloric diet, Nutrigenetics, PPAR-γ2

## Abstract

***Purpose:*** Obesity is a multi-factorial health problem which results from the interaction of environmental and genetic factors. The aim of the present study was to determine the effects of dried licorice extract with a calorie restricted diet on anthropometric indices and insulin resistance with nutrigenetic approach.

***Methods:*** For this pilot, double-blind, placebo-controlled randomized clinical trial, 72 eligible subjects were randomly allocated to Licorice or placebo group. They received a low-calorie diet either with a 1.5 g/day of Licorice extract or placebo for 8 weeks.

***Results:*** There were no significant differences in anthropometric indices and dietary intake in genotype subgroups at the baseline. Findings indicated that supplementation with Licorice extract did not change anthropometric indices and biochemical parameters significantly compared to a hypocaloric diet alone. However, from the nutrigenetic point of view, significant changes in anthropometric indices and QUICKI were observed in the Pro12Pro genotypes compared to the Pro12Ala at the end of the study (p<0.05 in all variables). Moreover, no interactive effect of the Licorice supplement and Pro12Ala genotype was found.

***Conclusion:*** In obese subjects, the Pro/Pro polymorphism of the PPAR-γ2 gene seems to induce favourable effects on obesity management. Further studies are needed to clarify whether PPAR-γ2 gene polymorphisms or other obesity genes can affect responses to obesity treatment.

## Introduction

*Glycyrrhiza glabra L*. (Fabaceae) generally which known as Licorice is a medicinal herb that widely grows in Mediterranean region and the south-west Asia. It contains various components with pharmacological properties including glabridin, glycyrrhizin, beta-Glycyhrritinic acid, flavonoids, sterols, amino acids, chalcones, isoflavones and triterpenoidsaponins.^1,2^ Licorice root frequently used in traditional medicine particularly for gastric and duodenal ulcers, helicobacter pylori effects and allergenic reactions. Previous studies have reported antioxidant, anti-mutagenic, anti-inflammatory, anti-viral, anti-bacterial and anti-asthmatic properties for licorice and its components.^1,3^ Additionally in the resent years, the anti-obesity effects of Licorice and its effective ingredients have been reported.^4-9^

Obesity is a chronic metabolic disorder which defines as excessive or abnormal fat accumulation.^10^ Obesity is one of the greatest health threats and it can result in a number of chronic diseases including cardiovascular diseases, diabetes, dyslipidemia and some cancers.^10^ Due to a dramatic increase in obesity prevalence, researchers attempt to find effective medications or supplements for obesity management.^4^ Previous studies have demonstrated several side effects for anti-obesity biochemical medications.^11^ Therefore, tendency to using complementary therapies such as medicinal herbs is increasing.^11^ It has been suggested that Licorice can affect obesity and its complications including insulin resistance and lipid profile through various mechanisms.^12-14^ However, there are limited clinical trials with contrary results for anti-obesity properties of Licorice.^4,15-17^

Obesity is a multi-factorial health problem which results from the interaction among metabolic, physiological, social, behavioural, and genetic factors.^10^ Proliferator-activated receptor gamma-2 (PPAR-γ2) has been considered as a candidate gene for obesity phenotype and its complications.^18^ PPAR-γ gene, a type II nuclear receptor located on chromosome 3p-25 in humans. The PPAR-γ2 isoform is expressed exclusively in adipose tissue, and it plays a main role in adipogenic differentiation, lipogenesis, energy homeostasis and insulin sensitivity.^19,20^ Pro12Ala (rs1801282) is one of known single nucleotide polymorphisms (SNPs) of PPAR-γ2 gene. Following a missense mutation (CCA→ GCA), Proline is substituted to Alanine at codon 12 exon B and it can affect transcriptional activity of PPAR-γ2 gene.^21^ Some previous studies have indicated effective roles for Pro12Ala genotype in response to dietary interventions for obesity management.^22,23^

Since PPAR- γ2 gene polymorphism (Pro12Ala) is a common obesity candidate gene and its prevalence has been determined in Iranian population,^24^ we considered it for the present study. To the best of our knowledge, the effects of supplementation with licorice extract for obesity management have been evaluated in limited clinical trials. Moreover, it seems the effect of Licorice with respect to genetic differences and gene-diet interactions has not been evaluated so far. Therefore, the aim of the present study was to determine the effects of dried licorice extract with a calorie restricted diet on anthropometric indices and insulin resistance with nutrigenetic view point, considering the polymorphism of PPAR- γ2 (Pro12Ala) gene.

## Materials and Methods

### Subjects 

The present study was a part of a study on obesity gene polymorphisms with 216 sample size. The intervention section was conducted on 72 volunteer obese subjects in Tabriz, Iran. Iranian subjects aged 20–50 years old with a body mass index (BMI) equal or more than 30.0 kg/m^2^were recruited at Sheykhoraees Clinic from March to September 2012. The exclusion criteria were as follows: cardiovascular diseases, liver, thyroid and kidney disorders, diabetes, smoking, taking any anti-obesity medications, vitamin-mineral supplements, antioxidant medications and herbal drugs throughout 3 months ago, menopause, pregnancy and lactation.

At the beginning of the trial, all eligible volunteers signed a written consent form.

### Study design

This study was a pilot, double-blind, placebo-controlled randomized clinical trial. Based on the findings of a previous study on frequency of the PPAR-γ2 (Pro12Ala) gene polymorphism in Iranian obese subjects (30.8%),^24^ we recruited 216 obese subjects who referred to the obesity clinic and basically screened them for presence of Pro12Ala polymorphism. For getting additional information on the intervention, a 2:1 allocation rate was used.^25^ Finally, 72 subjects were selected based on this SNP, 24 Ala carriers (Pro/Ala genotype) and 48 non-Ala carriers (Pro/Pro).

The participants were randomly allocated to two groups using a block randomization procedure (random number table; block size=2). After matching the subjects based on sex, age and BMI, they were allocated into each arm of the trial. To maintain blinding, randomization and allocation was conducted by a subject with no involvement in the trial. In addition, the researchers and participants remained blind throughout randomization and allocation until data analysis. Both groups received a balanced calorie-restricted diet. A dietician designed an individualized diet that was explained in details in our previous study.^12^ Intervention and placebo group took 1.5 gr/day (three 500 mg capsules; one capsule 30 min before each main meal) of dried licorice extract and placebo (corn starch; three 500 mg capsules) for 8 weeks, respectively.

Both supplement and placebo were provided for the participants in similar opaque pockets. The colour and appearance of the capsules were the same in both groups. The subjects received the pockets during the first interview and every 20 days. After randomization, the supplements were distributed among the volunteers based on the allocation code (A or B). To determine compliance with the supplements, the participants were asked to return the pockets (empty or full) in each visit. Therefore, compliance could be estimated by counting the remaining capsules. The participants were warned to be excluded if they had taken less than 90% of the supplements through the intervention. Moreover, the participants were advised to continue their usual physical activities and to contact the researcher for any side effects related to taking the supplements. At baseline, demographic characteristics including age, family history of obesity, disease history, and medications was collected.

### Licorice extract characteristics

The licorice extract was prepared by Darook pharmaceutical company (Esfahan, Iran). The dried hydroalcoholic extract of licorice root (ethanol 70: water 30% v/v) contained lowered Glycyrrhizin (<0.01%). The yield of extraction was 10% (10gr extract/100 gr powdered licorice root).

### Data collection

#### Dietary intake, anthropometric indices and physical activity assessments

Dietary intake and anthropometric indices were measured at baseline and at the end of the study. Dietary intake was evaluated using a three-day (two weekdays and one weekend) 24-h food recall. Necessary information about how to estimate and record daily food intake were presented. It was analyzed using the Nutritionist IV software (First Databank Inc., Hearst Corp., San Bruno, CA) modified for Iranian foods for total energy and macronutrients. Weight, height, waist circumference and hip circumference were measured using standard methods as have been explained elsewhere.^12^ Body mass index (BMI) was calculated by dividing weight in kilogram by height in meters squared. Physical activity level was evaluated using International physical activity questionnaire (IPAQ)^26^ and then participants were categorized into moderate or sedentary physical activity groups.

#### Biochemical measurements

At baseline and at the end of the intervention, after 12-14h fasting, 10 mL blood samples were collected from each participant between 8:00 and 9:30 a.m. FBS levels was analyzed on the day of sampling and the remaining serum was stored at -80°C until assay time. FBS concentration was measured by the enzymatic method using an Abbot Model Aclyon 300, USA autoanalyzer with a kit from Pars-Azmon (Tehran, Iran). Insulin concentration was measured using the ELISA method with commercial kit (Monobind, Denmark). The quantitative insulin sensitivity check index (QUICKI) was calculated according to the following formula: QUICKI = [1/ ( log (Insulin) + log(FBS)].

#### Genetic Assessments

Venous blood samples (3mL) were collected and transferred into a Vacutainer tube containing EDTA. DNA was extracted from peripheral blood, on the basis of the Cinagen Kit dNp protocol (DNG plus DNA Extraction Kit, Sinagene Company, Tehran, Iran). To determine the SNPs, polymerase chain reaction (PCR), followed by restriction fragment length polymorphism (RFLP) assays was used.

The sequences of PCR primers for Pro12Ala PPARγ2 variant (rs1801282) were as follows:

Forward primer: 5´-TCTGGGAGATTCTCCTATTGGC-3’

Reverse primer: 5´- CTGGAAGACAAACTACAAGAG-3’

PCR premix consisted of PCR buffer 10 mM, dNTP 0.2 mM, Mgcl 1.5 mM, each primer (0.8 pM/μl) with 1 U Tag polymerase. Cycling was performed in a thermal cycler as: 95 °C for 4 min, 94 °C for 1 min, 58 °C for 1min, Go to 2 for 38 cycles, 72 °C for 5 min. Then, the PCR product was digested with HhaI restriction enzyme for 1.5 h at 37 °C using 2 μl Buffer tango (10×) and HhaI 0.5μl (Fermentas, Lithuania). The digested products were analyzed by electrophoresis in a 2.5% agarose gel stained with ethidium bromide that was exposed under UV to visualize the fragments. Genotyping was repeated in all Pro12Ala heterozygotes and randomly selected Pro12 homozygotes; their reproducibility was 100%.

#### Statistical analysis

The Kolmogorov-Smirnov test was used for evaluating the normality of the data distribution. The results are reported as mean±SD and Median (25^th^, 75^th^) for data with normal and non-normal distribution, respectively. For comparison data and detection the differences among the study groups, one-way ANOVA (all the variables except insulin) and Kruscal-Wallis H (for insulin) test was used at the beginning and at the end of the trial. The effect of supplement, Pro12Ala gene polymorphism and gene-supplement interactions on changes of anthropometric indices and biochemical parameters was examined using two-way ANOVA test. Statistical analyses of all data were performed using SPSS version 13.0 (SPSS, Chicago, IL, USA), and p<0.05 was considered statistically signiﬁcant.

## Results

**Figure 1:**
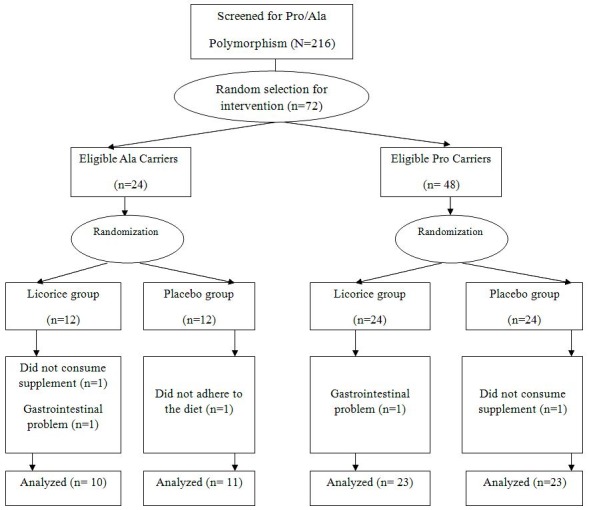
Summary of the randomization, allocation and follow-up of participants in the study groups

A total of 216 obese subjects were screened for the PPARγ (Pro12Ala) gene polymorphism. The prevalence rates were 74.1% (n=160) for Pro/Pro and 25.9% (n=56) for Pro/Ala. Moreover, no participants had the Ala/Ala genotype. Throughout the trial, three subjects in the Licorice group were excluded, because of gastric complications (n=2) and not adhering to the study procedure (n=1). In the placebo group, two subjects were also excluded due to not adhering to the study procedure and gastrointestinal disorder (Figure 1). Finally 67 subjects completed the study. The capsule counts indicated that all the participants who completed the study had high compliance (>90%) with the supplementation.

The baseline characteristics of participants based on the Pro12Ala polymorphism are indicated in Table 1. No significant differences were observed in the study population at baseline. As shown in Table 2, there were no significant differences in dietary intakes of the comparable groups at the beginning of the trial. At the end of the study, only differences in energy intake among the study groups stratified by the Pro12Ala polymorphism were significant (p<0.01) (Table 2). Reduction in the energy intake of subjects with Pro/Pro genotype who received licorice supplement was significantly more than the Pro/Ala group (-48.0% vs. -33.4%). Table 3 illustrates the effects of supplement, Pro/Ala polymorphism and the gene-supplement interactions on subgroups of PPAR-γ2 gene in the licorice and placebo group. Findings indicated that supplementation with Licorice extract did not change anthropometric indices and biochemical parameters significantly compared to a hypocaloric diet alone. But dependent on the PPAR-γ2 polymorphism, significant changes in body weight, BMI, WC, WHR and QUICKI were observed at the end of the study. Moreover, no interactive effect of licorice supplementation and PPAR-γ (Pro12Ala) was found on anthropometric indices, serum levels of FBS, insulin, and QUICKI (Table 3).

**Table 1:** Anthropometric indices and biochemical parameters of the participants in the Licorice and placebo group based on Pro12Ala genotype at the baseline

**-**	**Licorice Group (n=33)**		**Placebo Group (n=34)**		**P-value†**
**Variables**	Pro/Pro	Pro/Ala	Pro/Pro	Pro/Ala	-
**Age (years)**	37.4 ±14.7*	36.0±8.7	41.8±13.8	42.7±9.1	0.9
**Weight (kg)**	89.8±18.4	86.3±11.3	83.2±9.8	79.9±5.9	0.3
**BMI(kg/m** ^ 2 ^ **)**	34.3±5.2	32.9±2.0	33.4±3.3	33.3±2.3	0.4
**Waist Circumference (cm)**	109.2±14.0	101.2±10.2	110.3±11.0	106.1±9.0	0.09
**Hip Circumference (cm)**	117.1±10.5	114.4±7.0	112.3±9.3	113.2±6.9	0.6
**FBS(mg/dL)**	100±8.6	97.2±7.6	97.0±8.6	94.5±6.5	0.2
**Insulin**	9.0(5.3,13.2)‡	10.5 (7,14.5)	8.5(4.2,10.0)	9.0(4.7,16.6)	0.2**
**QUICKI**	0.34±0.03	0.34±0.02	0.35±0.02	0.35±0.04	0.8

BMI: Body mass index; FBS: Fasting blood sugar; QUICKI: Quantitative insulin sensitivity check index *Mean±SD ‡ Median (25^th^, 75^th^ ) †One-way ANOVA; Comparison of between-group differences at the baseline ** Kruskal-Wallis H

**Table 2:** Comparison of dietary intake based on the PPAR-γ2 genotype in the Licorice and placebo groups at the baseline and after the intervention

**Variables**	**At the baseline**				**P-value†**	**At the end**				**P-value†**
	**Licorice**		**Placebo**			**Licorice**		**Placebo**		
	Pro/Pro	Pro/Ala	Pro/Pro	Pro/Ala		Pro/Pro	Pro/Ala	Pro/Pro	Pro/Ala	
**Energy (kcal/day)**	2498±414*	2476±538	2326±382	2436±266	0.7	1297±227	1648±239	1339±230	1480±247	**<0.01**
**Carbohydrate (g/day)**	301.2±78.9	314.5±73.5	312.0±59.8	345.4±63.7	0.2	174.1±45.4	208.3±59.1	196.4±47.6	228.6±46.5	0.7
**Protein (g/day)**	84.9±33.7	109.5±43.6	97.8±20.2	94.9±19.9	0.2	41.9±11.6	39.3±14.6	43.4±7.1	42.2±12.2	0.4
**Total fat (g/day)**	105.3±45.1	86.6±31.4	89.8±29.8	93.3±37.7	0.9	41.3±15.0	48.0±23.2	42.2±13.2	44.1±14.3	0.3
**SFA (g/day)**	14.3±7.9	15.0±7.2	13.5±4.7	13.7±4.3	0.8	9.1±3.1	9.3±4.8	4.9±1.2	11.7±5.4	0.3
**MUFA (g/day )**	15.9±4.4	18.3±5.3	14.3±4.2	15.6±5.9	0.4	6.6±3.7	11.2±3.8	12.2±2.6	13.5±3.8	0.5
**PUFA (g/day )**	15.4±3.2	16.9±5.4	16.5±2.6	17.4±6.3	0.5	17.1±8.4	19.4±6.4	14.6±5.5	12.2±8.4	0.8
**Dietary Fiber (g/day)**	10.7±3.9	8.7±3.0	8.7±3.2	9.9±4.6	0.6	12.3±2.7	9.7±3.2	10.5±4.5	11.9±4.1	0.7

SFA: Saturated fatty acid; MUFA: Mono unsaturated fatty acid; PUFA: Poly unsaturated fatty acid *Mean±SD †One-way ANOVA: Comparison of between-group differences; **p<0.05 considered significant**

**Table 3:** The effects of supplementation and genotype and their interaction on anthropometric indices and biochemical parameters in each genotype in Licorice and placebo group

**Variables**	**At the baseline**				**At the end**				**P-value†**		
	**Licorice**		**Placebo**		**Licorice**		**Placebo**		**suppl‡**	**Geno-type††**	Intera-ction**
	Pro/Pro	Pro/Ala	Pro/Pro	Pro/Ala	Pro/Pro	Pro/Ala	Pro/Pro	Pro/Ala			
**Weight (kg)**	89.8±18.4	86.3±11.3	83.2±9.8	79.9±5.9	82.7±12.5	84.6±10.7	79.9±8.8	78.9±6.7	0.2	**0.03**	0.4
**BMI (kg/m** ^ 2 ^ **)**	34.3±5.2	32.9±2.0	33.4±3.3	33.3±2.3	31.8±4.1	32.2±2.0	32.1±2.6	32.9±2.6	0.2	**0.03**	0.4
**WC (cm)**	109.2±14.0	101.2±10.2	110.3±11.0	106.1±9.0	102.5±12.1	99.0±8.5	101.5±10.1	104.2±10.5	0.5	**<0.01**	0.5
**WHR**	0.93±0.08	0.86±0.10	0.98±0.09	0.94±0.09	0.91±0.08	0.85±0.11	0.92±0.11	0.93±0.10	0.5	**0.04**	0.4
**FBS (mg/dL)**	100±8.6	97.2±7.6	97.0±8.6	94.5±6.5	102.2±6.7	96.8±7.3	96.4±8.1	96.4±5.5	0.8	0.8	0.1
**Insulin**	9.0 (5.3,13.2)‡	10.5 (7,14.5)	8.5 (4.2,10.0)	9.0 (4.7,16.6)	6.8 (4.6,10.8)	8.1 (5.6,10.5)	4.0 (2.5,7.8)	6.7 (2.7,10.4)	0.7	0.2	0.08
**QUICKI**	0.34±0.03	0.34±0.02	0.35±0.02	0.35±0.04	0.35±0.04	0.35±0.02	0.38±0.04	0.34±0.02	0.9	**0.04**	0.1

BMI: Body mass index; WC: Waist circumference; WHR: Waist to hip ratio; FBS: Fasting blood sugar; QUICKI: Quantitative insulin sensitivity check index *Mean±SD; † Two-way ANOVA: Comparison of changes in variables at the end of the study; ‡ The effect of Licorice supplement on outcome variable; †† The effect of PPARγ (Pro12Ala) on outcome variable;** The gene-supplement interaction on outcome variable

## Discussion

Our findings indicated that independent of the licorice supplementation, body weight, BMI, WC and WHR decreased and QUICKI increased in obese subjects with regard to the Pro12Ala polymorphism of PPAR-γ2 gene. In subjects with Pro/Pro genotype, more changes were observed at the end of the study. Furthermore, no interactions between gene and interventions were found.

There are limited clinical trials on anti-obesity effects of supplementation with Licorice or its active components. Tominaga *et al.* found that 300 and 1800 mg/day supplementation with Kaneka Glavonoid rich oil ^TM^ (LFO) suppressed weight gain in overweight subjects after 3 months. In another study by Tominaga *et al.,* 300 mg/day LFO decreased WC and visceral fat after 3 months.^4^ According to Armanina *et al.* study, 3.5 g/day Licorice root did not change BMI in normal weight subjects after 8 weeks. They suggested that positive effects of licorice may be due to its strong taste that can decrease food intake and appetite.^17^ Possible anti-obesity mechanisms for licorice are as follows: 1) regulation of lipid metabolism and lipolysis through effects on gene expression in fatty acid synthesis pathways and increase of fatty acid oxidation^5,2^) activation of PPAR-γ gene^5,3^) reduction in appetite due to strong taste ^17^ and 4) reduction in fat intestinal absorption.^6^

However, two clinical trials have not shown the anti-obesity effects of licorice. Bell *et al.* reported that Glavonoid^TM^(Licorice Flavenoid Oil (LFO)) did not reduce body weight and WC in overweight and grade I-II obese subjects after 8 weeks.^16^ Hajiaghamohammadi *et al.* also reported that 2 g/day aqueous licorice extract did not reduce BMI in patients with non-alcoholic fatty liver disease after 8 weeks.^15^ Discrepancy in findings might be related to differences in individual’s characteristics, study design, dosage and type of licorice supplement, disease background, dietary intake, physical activity level, duration of the intervention and genotypes.

In our study, we compared the efficacy of licorice supplement in combination with a low-calorie diet vs. a low-calorie diet alone with regard to the Pro12Ala polymorphism. It seems that no clinical trial has evaluated the licorice-gene interactions in obesity management. But in some previous studies, the effects of weight-loss diet on subjects with respect to the Pro/Ala genotype were evaluated. In line with our study, Vogel et al., indicated that following a 6-wk very low calorie diet (VLCD), weight reduction in overweight/ obese subjects with Pro12Pro genotype was more than Pro12Ala genotype.^27^But Delahanty et al., demonstrated that independent of intervention (metformin intake and lifestyle changes), weight reduction in pre-diabetic obese subjects with Pro/Ala genotype was more than homozygote subjects after 6 months.^22^ Based on Goyenechea et al's study, the Pro12Ala genotype was also more frequently reported in Spanish subjects with successful weight maintenance after 10 weeks of dietary intervention.^28^ Curti et al., showed that lifestyle modifications decreased body weight in subjects with high risk of cardiovascular diseases independent of the PPAR-γ2 polymorphism.^29^ In our study, no interactions between PPAR-γ2 and licorice supplementation were observed. Anthropometric indices were decreased in the homozygote subjects for Pro more than heterozygotes independent of the intervention. Differences in race, study design, intervention, gene-gene interactions and gene –intervention interactions might lead to different findings.

In the present study, irrespective to the intervention, a significant difference in QUICKI was observed in genotype subgroups at the end of the study. Increase of QUICKI was more in subjects with Pro/Pro genotype compared to the Pro/Ala genotype. Limited clinical trials evaluated the effects of licorice on glycemic status. Tominaga *et al.* found that 1800 mg/day LFO did not change FBS and insulin concentrations in overweight subject after 12 weeks.^4^But Luan *et al.* indicated that 10 µM glabridin reduced insulin level and insulin resistance in women with polycystic ovary syndrome after 12 months.^30^ Based on Wu *et al.* study, glabridin decreased FBS and insulin resistance after 28 days in diabetic mice. They suggested that antioxidative property of glabridin might lead to anti-hyperglycemic effects of glabridin in diabetic model.^13^ Moreover in some studies, anti-diabetic effects of glycyrrihizin were reported.^31,32^ Glycyrrihizin can elevate PPAR-γ and glucose transporter 4 proteins in skeletal muscles and modulate glycemic status.^33,34^ In our study, owing to mineral corticoid actions and presser effects of Glycyrrihizin,^35^ Glycyrrihizin had been reduced to <0.01. This issue might result in no significant reduction in FBS and insulin concentrations. Moreover, disease background, BMI, dosages and form of licorice or its pure ingredients and the duration of intervention can involve in varying findings.

There are limited studies with contrasting findings on possible associations between the Pro12Ala and insulin response to calorie restriction. In contrary to our findings, Curti et al., reported that Pro12Ala polymorphism made no impact on glycemic status in responses to a low-calorie diet concurrent with exercise in Brazilians at high cardiometabolic risk.^29^ Stefanski et al. found no differences in insulin resistance and insulin secretion between the genotype groups in people with long-standing type 2 diabetes (BMI ≥30 kg/m^2^, a mean age of 64 years).^36^ But Garaulet et al. reported a protective role for the Ala12 allele against insulin resistance in a Spanish overweight/obese population who adhered to a Mediterranean diet and physical activity program for losing weight.^23^ Delahanty et al. also concluded that independent of the intervention, a significant association between the Ala12 allele at PPAR-γ was observed after short and long-term weight loss.^22^

In the present study, no participants had the Ala/Ala genotype, and previous studies have shown that Ala/Ala is found at zero frequency in many Asian populations.^37^ However, results would be more reliable if a larger sample size was studied. The main mechanism in the association between the Pro12Ala genotype and insulin sensitivity was not clear. However, the protective effect of a functional variant against insulin resistance was probably due to a reduction in transcriptional activity of PPAR-γ2 by activating a ligand-independent domain in the N-terminal. The location of the Pro12Ala substitution in the N-terminal region means that it can be involved in reducing transcription, as well as its association between genotypes and increased insulin activity.^22^

The present study had some limitations. Sample size was relatively small and we did not find the Ala12Ala polymorphism in our study population. However, its frequency has often been reported as being too low in other populations. Moreover in the present study, other polymorphisms of the PPAR-γ2 gene were not evaluated. The strengths of the current study are as follows: it was double blinded and gene/intervention interaction was examined. Furthermore, the measured parameters were adjusted for some known confounding factors.

## Conclusion

In Iranian obese subjects, it seems the Pro/Pro polymorphism of the PPAR-γ2 gene induce favourable effects on obesity management and insulin sensitivity. Furthermore, our findings did not support greater benefits for licorice supplementation vs. a low-calorie diet alone. Further clinical trials are needed to clarify whether PPAR-γ2 polymorphism or other obesity gene polymorphism can affect responses to obesity treatment.

## Acknowledgments

We are grateful to the participants for their cooperation. The authors also would like to thank Drug Applied Research Center, Tabriz University of Medical Sciences for funding of the project.

## Ethical Issues

The present study was approved by the Ethical Committee of Tabriz University of Medical Sciences and it was registered on the Iranian Registry of Clinical Trials (IRCT registration number: IRCT2013062811288N3).

## Conflict of Interest

The authors declare no conflict of interests.
